# Complete Genome Sequence of a Moderately Virulent *Aeromonas hydrophila* Strain, pc104A, Isolated from Soil of a Catfish Pond in West Alabama

**DOI:** 10.1128/genomeA.00554-14

**Published:** 2014-06-05

**Authors:** Julia W. Pridgeon, Dunhua Zhang, Lee Zhang

**Affiliations:** a Aquatic Animal Health Research Unit, USDA, ARS, Auburn, Alabama, USA; b Genomics and Sequencing Laboratory, Department of Entomology and Plant Pathology, Auburn University, Auburn, Alabama, USA

## Abstract

*Aeromonas hydrophila* pc104A is a moderately virulent strain isolated from the soil of a catfish pond in west Alabama in 2010. Its full genome is 5,023,829 bp. The availability of this genome will allow comparative genomics to identify the virulence genes that are important for pathogenesis or immunogens for the purpose of vaccine development.

## GENOME ANNOUNCEMENT

The Gram-negative bacterium *Aeromonas hydrophila* is the causative agent of motile aeromonad septicemia (MAS) (1), also known as epizootic ulcerative syndrome (2). Typical symptoms of MAS in fish include red sores, necrosis, ulceration, and hemorrhagic septicemia (3). In west Alabama, MAS disease outbreaks have led to an estimated annual loss of >3 million pounds of food-sized channel catfish since 2009 (4–8). The complete genome of highly virulent isolates of *A. hydrophila* ML09-119 and *A. hydrophila* AL09-71 from the western Alabama MAS disease outbreak were recently published (8, 9). Lateral gene transfer has been implicated as the molecular basis of the emergence of an *A. hydrophila* epidemic outbreak (10). Strain *A. hydrophila* pc104A was isolated from the soil of catfish pond in west Alabama in 2010. Virulence studies revealed that *A. hydrophila* pc104A was at least 1,000-fold-less virulent than *A. hydrophila* AL09-71 based on estimated 50% lethal dose (LD_50_) values. It was unknown whether the less virulent *A. hydrophila* pc104A and the highly virulent isolates, such as *A. hydrophila* AL09-71 or *A. hydrophila* ML09-119, were different at the genomic DNA level. Therefore, the complete genome sequence of *A. hydrophila* pc104A was determined in this study.

The genome of *A. hydrophila* pc104A was sequenced using the Illumina 1500 HiSeq platform. BioNumerics (Applied Maths) was used to assemble a total of 23,280,526 sequence reads, with an average length of 100.18 bp (estimated 464× coverage). Using the west Alabama epidemic isolate *A. hydrophila* ML09-119 genome (accession no. CP005966) as a reference, the assembled genome of *A. hydrophila* pc104A is 5,023,829 bp, with a G+C content of 60.8%. RNAmmer (11) predicted 11, 10, and 10 copies of 5S RNA, 16S RNA, and 23S RNA, respectively, in the genome of *A. hydrophila* AL09-71, which is similar to those in the genomes of *A. hydrophila* ML09-119 (8) or *A. hydrophila* AL09-71 (9). The RAST server (12) predicted 4,492 genes belonging to 534 subsystems, including 466 involved in carbohydrate metabolism, 437 in amino acids and derivatives, 295 in cofactors, vitamins, prosthetic groups, or pigments, 263 in protein metabolism, 224 in RNA metabolism, 189 in cell wall and capsule synthesis, 183 in membrane transport, 172 in respiration, 159 in stress response, 136 in fatty acid and lipids synthesis, 127 in nucleosides and nucleotides, 127 in motility and chemotaxis, 124 in DNA metabolism, 100 in regulation and cell signaling, 89 in virulence, disease, and defense, and 19 in the subsystem of phages, prophages, transposable elements, and plasmids.

### Nucleotide sequence accession number.

The complete genome sequence of *A. hydrophila* pc104A was deposited at GenBank under the accession no. CP007576.

## References

[1] Harikrishnan R Rani MN Balasundaram C 2003 Hematological and biochemical parameters in common carp, *Cyprinus carpio*, following herbal treatment for *Aeromonas hydrophila* infection. Aquaculture 221:41–50. 10.1016/S0044-8486(03)00023-1

[2] Mastan SA Qureshi TA 2001 Role of bacteria in the epizootic ulcerative syndrome (EUS) of fishes. J. Environ. Biol. 22:187–192 12017259

[3] Karunasagar I Rosalind GM Karunasagar I Gopal Rao K 1989 *Aeromonas hydrophila* septicaemia of Indian major carps in some commercial fish farms of West Godavari District, Andhra Pradesh. Curr. Sci. 58:1044–1045

[4] Hemstreet B 2010 An update on *Aeromonas hydrophila* from a fish health specialist for summer 2010. Catfish J. 24: 4

[5] Pridgeon JW Klesius PH 2011 Molecular identification and virulence of three *Aeromonas hydrophila* isolates cultured from infected channel catfish during a disease outbreak in West Alabama (USA) in 2009. Dis. Aquat. Organ 94:249–253. 10.3354/dao02332 21790073

[6] Pridgeon JW Klesius PH 2011 Virulence of *Aeromonas hydrophila* to channel catfish *Ictaluras punctatus* fingerlings in the presence and absence of bacterial extracellular products. Dis. Aquat. Organ 95:209–215. 10.3354/dao02357 21932532

[7] Griffin MJ Goodwin AE Merry GE Liles MR Williams MA Ware C Waldbieser GC 2013 Rapid quantitative detection of *Aeromonas hydrophila* strains associated with disease outbreaks in catfish aquaculture. J. Vet. Diagn. Invest. 25:473–481. 10.1177/1040638713494210 23847227

[8] Tekedar HC Waldbieser GC Karsi A Liles MR Griffin MJ Vamenta S Sonstegard T Hossain M Schroeder SG Khoo L Lawrence ML 2013 Complete genome sequence of a channel catfish epidemic isolate, *Aeromonas hydrophila* strain ML09-119. Genome Announc. 1(5):e00755-13. 10.1128/genomeA.00755-13 24051325 PMC3778208

[9] Pridgeon JW Zhang D Zhang L 2014 Complete genome sequence of the highly virulent *Aeromonas hydrophila* AL09-71 isolated from diseased channel catfish in west Alabama. Genome Announc. 2(3):e00450-14. 10.1128/genomeA.00450-14 24855300 PMC4031339

[10] Hossain MJ Waldbieser GC Sun D Capps NK Hemstreet WB Carlisle K Griffin MJ Khoo L Goodwin AE Sonstegard TS Schroeder S Hayden K Newton JC Terhune JS Liles MR 2013 Implication of lateral genetic transfer in the emergence of *Aeromonas hydrophila* isolates of epidemic outbreaks in channel catfish. PLoS One 8:e80943. 10.1371/journal.pone.0080943 24278351 PMC3835674

[11] Lagesen K Hallin P Rødland EA Staerfeldt HH Rognes T Ussery DW 2007 RNAmmer: consistent and rapid annotation of ribosomal RNA genes. Nucleic Acids Res. 35:3100–3108. 10.1093/nar/gkm160 17452365 PMC1888812

[12] Aziz RK Bartels D Best AA DeJongh M Disz T Edwards RA Formsma K Gerdes S Glass EM Kubal M Meyer F Olsen GJ Olson R Osterman AL Overbeek RA McNeil LK Paarmann D Paczian T Parrello B Pusch GD Reich C Stevens R Vassieva O Vonstein V Wilke A Zagnitko O 2008 The RAST server: Rapid Annotations using Subsystems Technology. BMC Genomics 9:75. 10.1186/1471-2164-9-75 18261238 PMC2265698

